# Cryopreservation of Cell Sheets for Regenerative Therapy: Application of Vitrified Hydrogel Membranes

**DOI:** 10.3390/gels9040321

**Published:** 2023-04-10

**Authors:** Yoshitaka Miyamoto

**Affiliations:** 1 Department of Reproductive Biology, National Research Institute for Child Health and Development, Setagaya-ku, Tokyo 157-8535, Japan; miyamoto-ys@ncchd.go.jp or myoshi1230@gmail.com; Tel.: +81-3-3416-0181; 2 Department of Maternal-Fetal Biology, National Research Institute for Child Health and Development, Setagaya-ku, Tokyo 157-8535, Japan; 3 Graduate School of BASE, Tokyo University of Agriculture and Technology, Koganei, Tokyo 184-8588, Japan; 4 Department of Mechanical Engineering, Tokyo Institute of Technology, Meguro-ku, Tokyo 152-8552, Japan

**Keywords:** cell sheet, cryopreservation, vitrified hydrogel membrane, collagen, regenerative therapy, cell therapy

## Abstract

Organ transplantation is the first and most effective treatment for missing or damaged tissues or organs. However, there is a need to establish an alternative treatment method for organ transplantation due to the shortage of donors and viral infections. Rheinwald and Green et al. established epidermal cell culture technology and successfully transplanted human-cultured skin into severely diseased patients. Eventually, artificial cell sheets of cultured skin were created, targeting various tissues and organs, including epithelial sheets, chondrocyte sheets, and myoblast cell sheets. These sheets have been successfully used for clinical applications. Extracellular matrix hydrogels (collagen, elastin, fibronectin, and laminin), thermoresponsive polymers, and vitrified hydrogel membranes have been used as scaffold materials to prepare cell sheets. Collagen is a major structural component of basement membranes and tissue scaffold proteins. Collagen hydrogel membranes (collagen vitrigel), created from collagen hydrogels through a vitrification process, are composed of high-density collagen fibers and are expected to be used as carriers for transplantation. In this review, the essential technologies for cell sheet implantation are described, including cell sheets, vitrified hydrogel membranes, and their cryopreservation applications in regenerative medicine.

## 1. Introduction

**Figure 1:**
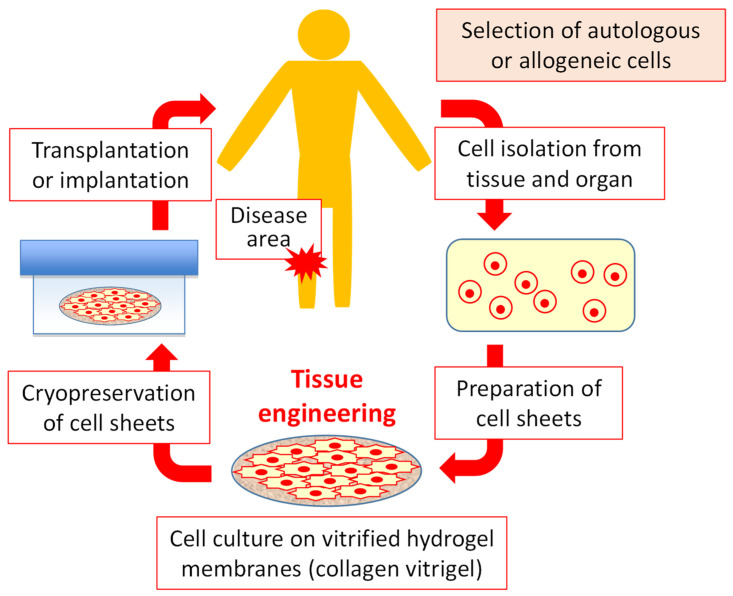
Regenerative medicine concept of cell sheets.

Organ transplantation is the first and most effective treatment when a tissue or organ is missing or damaged. However, there is a need to establish an alternative treatment method for organ transplantation due to the shortage of donors and viral infections. As an alternative, Rheinwald and Green et al. established epidermal cell culture technology [1,2] and successfully transplanted human-cultured skin into severely diseased patients [3]. In other words, this was the beginning of regenerative medicine. Then, combining the fields of medicine and engineering, Langer, R., and Vacanti, J.P. proposed “tissue engineering” to regenerate organs by drawing out the regenerative ability of cells [4]. In “tissue engineering,” cells, scaffolds, and growth factors play significant roles [5], and cell sheets such as cultured skin can be created using these key factors. For example, cell monolayer sheets have been successfully fabricated using thermoresponsive polymers [6] and applied clinically to myocardial tissues [7]. In addition, focusing on scaffolds, the extracellular matrix (ECM) is a non-cellular constituent of all tissues and organs [8,9]. The major fibrous proteins were collagen, elastin, fibronectin, and laminin. Collagen is a major structural component of basement membranes and tissue scaffold proteins. Therefore, collagen is widely used in tissue engineering and regenerative medicine to process hydrogels and membrane structures. In particular, collagen hydrogel membranes (collagen vitrigel), created from collagen hydrogels through a vitrification process, are composed of high-density collagen fibers [10] and are expected to be used as carriers for transplantation. Furthermore, it is necessary to provide large-volume quality-controlled cell sheets for widespread medical applications. Here, I introduce the advances in cryopreservation that can provide a stable supply of cell sheets for regenerative therapy (Figure 1): 1. Targeted autologous or allogeneic cells are isolated to treat diseases. 2. Cell sheets are prepared and cryopreserved using a hydrogel membrane. 3. The frozen cell sheets are thawed and transplanted into diseased patients.

In this review, the essential technologies for cell sheet implantation are described, including cell sheets, vitrified hydrogel membranes, and their cryopreservation applications in regenerative medicine.

## 2. The History of Tissue Culture and Tissue Engineering

Harrison, R.G. (1907) observed the growth of nerve fibers in the embryonic tissue fragments of frogs removed from the body [11]. This was the beginning of the proof of the concept of “tissue culture” from animal tissue culture. Tissue culture is used to maintain, culture, and revive (regenerate) tissues, organs, or cells. The concept of “cell culture,” which is widely used in the world today, is thought to have started when Rous and Jones (1916) succeeded in growing and culturing free cells by trypsin treatment of animal tissues [12]. The world’s oldest mouse-derived L cell line [13,14] and human-derived HeLa cell line [15] were established and cell culture technology has rapidly developed. The tissue culture method of Enders et al. (1949) proved that polioviruses can be grown in cells derived from human tissues, including human connective tissue, intestine, liver, and kidneys [16]. Enders, J.F., Weller, T.H., and Robbins, F.C. won the Nobel Prize in Physiology in 1954 for their “Tissue Culture of Poliovirus”. With the development and production of poliovirus vaccines [17,18,19], synthetic culture media such as 199 medium [20], Eagle’s medium [21], and Dulbecco’s medium [22] have been developed, and stable two-dimensional cultures have become possible.

In contrast, three-dimensional culture began with van Wezel’s (1967) technique of agitating cells by attaching them to microcarrier supports [23] and Knazek et al.’s (1972) technique of perfusion culture of cells in hollow fiber supports [24], which aimed at high-density culture. Eventually, not only a three-dimensional environment but also an environment that more closely mimics the in vivo environment was created by utilizing the ECM. For example, Kleinman et al. (1986) used a mouse tumor tissue extract (Matrigel) to create basement membrane-like structures [25]. Bell et al. (1979) used collagen gels to create artificial dermal tissue [26] and artificial skin tissue [27]. From 1970 to 1990, culture techniques for tissue and organ regeneration using biomaterials, such as biological materials and synthetic polymers, spread rapidly. Eventually, Langer and Vacanti (1993) proposed “tissue engineering” to develop organ and tissue substitutes that enable the regeneration, maintenance, and repair of vital functions [4].

## 3. Regenerative Medicine and Cultured Human Skin

Conventional medicine promotes the healing of damaged tissues and organs through pharmaceutical and surgical treatments.

Regenerative medicine involves the repair and regeneration of lost tissue and organ functions using patient cells, other cells, or artificial tissue. Specifically, this refers to medical treatments that regenerate lost functions:

1. Stem cells and other cells are artificially cultured outside the patient’s body.

2. Tissue is artificially constructed from stem cells and other cells outside the patient’s body.

3. Devices incorporating living cells activate and differentiate intrinsic stem cells with cell growth differentiation factors.

Regenerative medicine has the potential to provide novel treatment options for previously untreatable diseases. Cells used in regenerative medicine include somatic cells (skin cells, muscle cells, etc.), which comprise the body, somatic stem cells, embryonic stem cells (ES), and induced pluripotent stem (iPS) cells. In the 1970s, epidermal cell culture technology was established by Rheinwald and Green et al. [1,2]. In 1981, they succeeded in cultured human skin transplantation for patients with severe diseases, which marked the beginning of regenerative medicine [3].

The cultured human skin was then used as a cell sheet in which human epidermal cells were cultured and grown in a medium containing fetal bovine serum, using mouse 3T3 cells (fibroblasts) as a support cell layer (feeder cells). The key cell culture technology involves the use of feeder cells. The technique is as follows: (1) a co-culture of human epidermal cells and irradiated mouse 3T3 cells to selectively proliferate human epidermal cells; (2) irradiation causes 3T3 cells to lose proliferative ability but maintains cell adhesion and promotes the proliferation of human epidermal cells; and (3) the presence of 3T3 cells suppresses the proliferation of human fibroblasts, which are mixed with each cell. In the United States, the cultured epidermal autografts (Epicel^®^, Genzyme Corporation: Boston, MA, USA, 1987) and the autologous cultured chondrocytes (Carticel^®^, Genzyme Corporation: Boston, MA, USA, 1997) received authorization from the Food and Drug Administration (FDA). About 20 years later, the cultured epidermal autograft (JACE^®^, Japan Tissue Engineering Co., Ltd.: Aichi, Japan, 2007) received authorization for Japan’s first regenerative medical products (cellular and tissue-based products). Currently, tissue-engineering technology for preparing cells and tissues for transplantation is essential for cellular and regenerative medicine.

## 4. Hydrogel and Artificial Cell Sheets

ECM hydrogels (fibrous proteins such as collagen, elastin, fibronectin, and laminin, and polysaccharides such as hyaluronic acid and proteoglycans), synthetic polymer hydrogels, and rigid polymer materials (polystyrene) have been used as scaffold materials for the preparation of cell sheets such as cultured epidermal autografts [10,25,26,27,28]. Okano et al. [6] used cell sheets cultured on a temperature-responsive polymer that detached in sheet form upon temperature changes as carriers for transplantation. Medical treatments using this technology have been developed for several tissues and organs, including cardiomyocytes [7]. Takezawa et al. developed a cell culture carrier (collagen vitrigel) that closely resembled the density of collagen fibers in vivo [10] and applied it to drug discovery research and regenerative medicine [29,30].

### 4.1. Extracellular Matrix (ECM)

The ECM is a non-cellular constituent of all tissues and organs [8,9]. Major fibrous proteins such as collagen, elastin, fibronectin, and laminin, polysaccharides such as hyaluronic acid, and proteoglycans are known components of the ECM. These biopolymers form fibrous or net-like structures, or hydrogels containing many water molecules. ECM serves as a physical scaffold for cells and provides the signals required for tissue morphogenesis, differentiation, and homeostasis [9].

#### 4.1.1. Collagen and Gelatin

Collagen is the major protein component of connective tissue and basement membranes and exists in numerous forms (Types I–XVIII) with varying tensile strengths and tissue distributions [31]. Stiffness, flexibility, and structural changes in many body tissues are caused by changes in collagen composition, cell restriction, and compartmentalization. Collagen in vivo consists of rigid triple helical structures of three molecular collagen chains that aggregate to form nanometer-scale collagen fibrils, forming hierarchically ordered higher-order structures [32]. Collagen extracted from living organisms is insoluble in water due to the presence of hydrophobic amino acid residues outside the collagen fibrils. Atelocollagen, prepared by hydrolyzing collagen by acid treatment, dissolves in an acidic aqueous solution as the collagen fibrils dissociate, while maintaining the triple-helical structure. When an acidic solution of atelocollagen is neutralized and maintained at 37 °C, the collagen fibrils aggregate into a network due to hydrophobic interactions to form a hydrogel. The resulting collagen hydrogel is widely used as a base material for cell adhesion and implantation owing to its high biocompatibility and physiological activity [33,34,35,36].

Gelatin is a denatured form of collagen, the main component of connective tissues such as the skin, bones, and tendons. The main chemical component is a linear amino acid polymer. Gelatin is denatured by (1) acid and heat treatment and (2) alkali treatment. The alkali treatment method produces more carboxyl groups than the acid and heat treatment method [33]. When heated, gelatin exists in a sol state (randomly coiled molecular structure). When the solution is cooled, some gelatin molecules change to their original collagen-like helical structure (triple-helical structure), forming a network that eventually loses fluidity and becomes a gel. This gel undergoes a reversible sol-gel structural change upon heating and cooling. Gelatin hydrogels have high biocompatibility and oxygen permeability. Nutrients are transported through water diffusion via the hydrogel. Additionally, cells can be incorporated into gelatin hydrogels. However, owing to their low mechanical strength, researchers have attempted to improve the strength of these materials [37]. The resulting gelatin hydrogel, similar to the collagen hydrogel described above, is widely used as a base material for cell adhesion and regenerative medicine [38].

#### 4.1.2. Elastin

Elastin is a protein of the ECM involved in the elastic recoiling ability. Elasticity is the property of “elastic fibers” that return to their original state when force is removed. In addition to its elastic recoil, elastin is biocompatible because it is chemically inert and can be used as a hydrogel [39,40]. It mainly forms fibrous elastic tissues in the skin, blood vessels, and ligaments and provides elasticity to tissues [41]. However, biogenic elastin is highly crosslinked and insoluble. Therefore, it is difficult to obtain elastin as a homogeneous and easily handled material. Specific amino acid repeat sequences are evident in the properties of elastin, with Val-Pro-Gly-Val-Gly (VPGVG) being the most abundant in elastin [42,43,44]. Elastin-like peptides (ELPs) based on this repeating sequence exhibit a reversible phase transition called inverse temperature transition (ITT) in water. Monomer ELP genes are synthesized by ligating double stranded oligonucleotide cassettes or in pUC19 [45]. As the temperature changes, the ELP exhibits hydrophobic properties above the phase transition temperature (Tt) and changes to hydrophilic properties below Tt. Because of their excellent self-assembly and high biocompatibility, ELPs are expected to be used in medical engineering applications, such as DDS and ECM scaffold materials. Sugawara-Narutaki et al. created a novel block ELP by combining two types of sequence motifs derived from elastin and reported that it self-assembled upon temperature stimulation in water to form a fiber structure (nanofibers) similarly to elastin derived from organisms [46]. Furthermore, hydrogels were obtained with 0.3 wt % ELP, and uniform nanofibers were successfully formed. Because elastin is highly hydrophobic, heterogeneous, and prone to aggregation, it is difficult to create uniform gels, and there are few reported cases [47,48,49]. In the future, elastin-like hydrogels are expected to be used as carriers for implantation in cellular and regenerative medicine.

#### 4.1.3. Fibronectin

Fibronectin (FN) is a large glycoprotein and cell-adhesion molecule [50]. Cellular fibronectin is present in many tissues, including the spleen, lymph nodes, blood vessel walls, liver, kidney, muscle, skin, brain, and peripheral nerves. The interaction between FN and cell surface receptors such as integrins promote cell adhesion, shape, migration, growth, and differentiation in vitro [51]. They have a variety of functions in vivo, including cell adhesion to the ECM and connective tissue formation and retention. In addition, the amino acid sequences of FN and vitronectin contain many portions in which arginine (Arg), glycine (Gly), and aspartic acid (Asp) form a continuous motif that binds to integrin molecules on various cell membranes and promotes cell adhesion and survival.

Recently, Trujilloa et al. reported the development of FN-based 3D hydrogels of controlled stiffness and degradability [52]. By incorporating vascular endothelial growth factor (VEGF) into FN-based 3D hydrogels, it is expected to be a useful implantation carrier in tissue engineering and regenerative medicine.

#### 4.1.4. Laminin

Laminin is a large protein that constitutes the basement membrane of the ECM [50]. It promotes the establishment and maintenance of multicellular systems and tissues, as well as cell adhesion, migration, and proliferation [51]. Laminin plays an essential role in the formation of the basement membrane and conferring cell adhesion. The cell adhesion activity of laminin, mediated by cell surface integrins, is extremely strong compared with that of other cell adhesion proteins. Therefore, laminin is attracting attention as a feeder-free culture substrate for human embryonic stem cells (hESCs) and induced pluripotent stem cells (hiPSCs) [53]. Human laminin-511 supports the stable culture of hESCs and hiPSCs [54]. Laminin is an important constituent in neuronal tissue and brain [55]. To create three-dimensional neuronal models with neurons that are similar to those of living organisms, it is useful to incorporate laminin into hydrogels. Azide-modified laminin is conjugated to hyaluronan–poly(ethylene glycol) (HA:PEG) hybrid hydrogels. Encapsulated human neuronal cells demonstrate high viability and grow into cross-linked hyaluronan–laminin hydrogels.

#### 4.1.5. Hyaluronan (Hyaluronic Acid)

Hyaluronan (HA) is a polysaccharide consisting of N-acetylglucosamine and D-glucuronic acid (GlcNAcβ1-4GlcAβ1-3) linked on a linear chain [56]. It is widely distributed in vivo and plays important roles in the skin, cartilage, and eyes. HA mediates its activity in cellular signaling, wound repair, morphogenesis, and matrix organization [57,58,59]. HA has an extremely high molecular weight, ranging from 100,000 Da in the serum to 8,000,000 Da in the vitreous of the eye [60], with a minimum molecular weight of 411. As hyaluronan contains carboxyl and hydroxyl groups, it is easily chemically modified and can be used to create hydrogels with gelators. As an example, thiol, haloacetate, dihydrazide, aldehyde, tyramine, and Huisgen cycloaddition are chemically modified with hyaluronic acid. These modified HA monomers are used to create HA hydrogels by photopolymerization and electropolymerization reactions. Radical polymerization has been applied to the formation of HA hydrogels as an example of photopolymerization. In addition, HA hydrogels are dissolved by hyaluronidase, which is present in vivo and widely used as a culture substrate, 3D tissues, and carrier for transplantation [61].

#### 4.1.6. Alginic Acid

Alginic acid is a polysaccharide found in brown algae and other algae and is composed of β-D-mannuronate and α-L-guluronate in a β-1,4 bonded structure [62]. The characteristic feature of alginic acid is that when a divalent metal cation is added to an aqueous solution, the alginic acid molecules form an egg-box structure [63], and a hydrogel can be created [64]. Alginic acid is insoluble in water, but is extracted as a soluble salt, such as sodium alginate, and is used as a food additive. Alginate is able to form gels independently of temperature changes [62]. The formation of alginate gels can be achieved by ionic bonding with cations or acid deposition [65]. In other medical applications, alginate is used in fibrous gels (surgical threads), alginate salts (wound dressings), drug delivery, and tissue engineering [66]. Islet transplantation is an effective therapeutic modality to stabilize glycemic control in type 1 diabetes patients [67]. Islet encapsulation in an alginate hydrogel can immunosuppress, and maintain long-term patient survival.

#### 4.1.7. Synthetic Polymer

Polyethylene glycol (PEG) is a hydrophilic polymer with high biocompatibility. PEG has been used as a medical material for a long time. PEG-based materials are the most well-known DDS carriers made of PEG [68,69,70]. They can improve blood retention and enhance drug efficacy, but are not biodegradable or cell-adhesive. PEG is also expected to have potential as a carrier for transplantation, because it can incorporate bioactive molecules and other substances into its network to create functionalized hydrogels [71].

Polyvinyl alcohol (PVA) is a polymer obtained from polyvinyl acetate via alcoholysis, hydrolysis, and aminolysis [33]. An aqueous PVA solution was subjected to repeated freeze-thaw cycles to crystallize the molecular chains and form hydrogels [72]. PVA hydrogels are used as biomaterials in artificial cartilage and joints [73].

Polylactic acid (PLA) is a polyester that is used as a medical material for surgical sutures. PLA has D isomer (poly-D-lactic acid, PDLA), L isomer (poly-L-lactic acid, PLLA), and racemic forms depending on the structure of the monomer unit, which has a chiral carbon [74]. PLA synthesis requires control of conditions (temperature, pressure, and pH), and the use of catalysts [75]. PDLA and PLLA form a stereocomplex and aggregated to form a gel. PLA is a very safe material for living organisms because it is easily degraded by hydrolysis, and its degradation product is lactic acid. Therefore, PLA-based materials have been applied in orthopaedic regenerative engineering [76].

### 4.2. Thermoresponsive Polymer

Poly-N-isopropyl acrylamide (PNIPAm) is a well-known temperature-responsive polymer. PNIPAm undergoes a hydration/dehydration transition with a phase transition temperature of 32 °C as a boundary. To utilize the structural change in PNIPAm, a polymer layer is synthesized by graft polymerization of the N-isopropyl acrylamide monomer (NIPAm) on the surface of the culture substrate. The PNIPAm, which is strictly controlled on the surface of the culture substrate, shows hydrophobic properties at 37 °C and changes to hydrophilic properties at 32 °C. Cells adhere and proliferate on the surface of the culture substrate at 37 °C, but cells detach from the surface of the culture substrate and can be recovered at temperatures below 32 °C [6]. Tissue engineering using temperature-responsive polymers can be used to create and recover various cell sheets, including fibroblasts, epithelial cells, and cardiomyocytes. For example, cell sheets created from cardiomyocytes beat independently. When cardiomyocyte sheets are stacked on a fibrin gel, beating can be observed with the naked eye. Furthermore, thicker and more functional myocardial tissue was created by adding a vascular network to the cardiomyocyte sheet and increasing the number of stacked sheets [77]. According to previous reports, the clinical application of cardiomyocyte sheets has been successful. Cartilage is exhausted owing to trauma and aging, and many patients experience knee osteoarthritis. To treat these diseases, laminated autologous cartilage sheets have been created and various cytokines are expressed on these sheets to support tissue repair and regeneration. Other artificial cell sheets have been created using temperature-responsive polymers, such as corneal epithelial sheets [78], oral mucosal epithelial cell sheets [79,80], and periodontal tissue sheets [81], which are expected to have clinical applications.

### 4.3. Vitrified Hydrogel Membranes

Vitrification gradually removes free and bound water by drying, thereby converting the material into one with glass-like properties with excellent strength and transparency [82]. Takezawa et al. produced vitrified collagen hydrogel membranes (collagen vitrigels) composed of high-density collagen fibers via gelation, vitrification, and rehydration from a collagen hydrogel composed of conventional low-density collagen fibers [10]. The collagen vitrigel is a thin sheet with superior strength and transparency ranging from 10–100 µm in thickness, similar to the connective tissue in vivo. In addition to the ability to culture heterologous cells on both sides of the collagen vitrigel, they are permeable to proteins and drugs [29,30,83]. Numerous applications have been reported, including a corneal microtissue patch test [84]. Carriers such as collagen vitrigels induce bone regeneration by local and sustained delivery of bone morphogenetic protein-2 [85]. In addition, researchers have reported its use as an artificial corneal endothelial graft as a cell scaffold [86] and as an implantable material for the reconstruction of the cornea [87] and trachea [88].

Vitrification and rehydration can convert other vitrified hydrogel membranes into new stable physical states. However, there are only a few reports on gelatin, a chondroitin sulfate-polyethylene glycol (CS-PEG) adhesive, a collagen-based membrane (collagen vitrigel, CV) combination [89], and a bi-layered carboxymethyl cellulose-collagen vitrigel dual-surface adhesion-prevention membrane [90]. In addition, collagen-elastin (CE) membranes have been fabricated on polydimethylsiloxane (PDMS), and their Young’s modulus has been evaluated using atomic force microscopy (AFM) to design precise hydrogel membranes that mimic the ECM [91]. Precisely designed vitrified hydrogel membranes have new possibilities and have been developed as carriers for implantation.

## 5. Artificial Cell Sheets and Cryopreservation

### 5.1. Cryopreservation of Cellular and Tissue-Based Products and Simple Cell Suspensions

To generalize regenerative and cell medicine, it is necessary to provide therapeutic cells in large quantities with quality control [92]. Therefore, it is essential to establish a cryopreservation system that provides a stable cell supply. However, cellular damage can be triggered by ice crystal formation and dehydration within cells during freezing and thawing [93,94,95,96]. Therefore, to obtain high-quality cells, it is necessary to consider the freezing technique, composition of the cryopreservation solution, and thawing method [95,97,98]. In 1949, Polge et al. discovered that glycerol exhibits cryoprotective effects on human and avian sperm [99]. Ten years later, Lovelock et al. found that dimethyl sulfoxide (DMSO) was superior to glycerol in cell penetration and removal after thawing [100]. Therefore, it is now widely used as a mainstream cryopreservation solution [101].

Since 1998, the Food and Drug Administration (FDA: Silver Spring, MD, USA) has updated its Cellular & Gene Therapy Products guidelines [102]. In addition, the Pharmaceuticals and Medical Devices Agency (PMDA: Tokyo, Japan) [103] and European Medicines Agency (EMA, European Union) [104] updated their guidelines in the 2000s. Many mesenchymal stem cell (MSCs) products are available at clinicaltrials.gov [105]. Kymriah (tisagenlecleucel) and TEMCELL HS Inj. (MSCs) containing 10% to 7.5 vol% DMSO are used in clinical practice [80]. As these products are prone to side effects when administered as-is after freeze-thawing, the DMSO concentration should be reduced to 3–4%. Thus, the cytotoxicity of DMSO reduces cell survival due to mitochondrial swelling, membrane potential damage, and the production of reactive oxygen species [106].

In recent years, the shortage of donors in transplantation medicine has become increasingly serious, and it is essential to supply therapeutic cells to patients. Miyamoto et al. developed a cryopreservation solution for the transplantation of hepatocytes [107,108] and stem cells [109]. Similar to the cellular- and tissue-based products described above, we used a cryopreservation solution containing 10% DMSO. Furthermore, cell damage was reduced by combining it with other cryoprotectants (sericin and maltose). Yamatoya and Miyamoto et al. also reported that this cryopreservation solution was effective for differentiated neuronal cells [110].

### 5.2. Cryopreservation of Tissues and Cell Sheets

Cryopreservation of tissues results in significantly different cooling, warming, and dehydration responses compared with simple cell suspensions. These differences are due to differences in tissue structure and significant differences in the freezing and thawing methods [111]. Medical treatments such as bone and tendon treatments can be successfully performed without living cell components after freezing [112]. However, many medical treatments, such as those for the heart, require the maintenance of a live cell component after freezing. Therefore, freezing and thawing conditions and cryopreservation solutions must be designed to prevent ice formation inside and outside various tissues. Thus, vitrification is an effective ice-free cryopreservation technique [113].

A few therapeutic frozen cell sheets have been reported as cellular- and tissue-based products. To be approved as a cellular- and tissue-based product, frozen cell sheets must be scientifically evaluated in terms of chemistry, biophysics, toxicology, and cryobiology [93,111,112,113,114]. The critical evaluation criteria were cell viability within the cellular tissue sheet and the in vivo tissue function. The vitrification of various tissues has been reported [115,116,117,118], with articular cartilage being a good example [112]. However, in slow freezing, most chondrocytes die by ice formation and the ECM is damaged, which is a significant obstacle to clinical application [119,120]. Nevertheless, no difference was observed between fresh and vitrified cartilage during transplantation, and approximately 85% of the cellular metabolic activity was maintained [112,121]. Vitrification has also been reported to be effective in many small tissue structures such as spheroids [122], organoids [123], and encapsulated cells [124]. Cryopreservation of skin grafts has been used for several years. Each skin graft is banked in a manner desirable for long-term preservation, improving graft performance and safety, and reducing risks to the recipient [125].

The preparation and cryopreservation of cell sheets covers a variety of tissues, including epithelial sheets [126,127,128,129,130], chondrocyte sheets [131,132], myoblast [133], stem cell [134,135,136] and other cell sheets [137,138,139] (Table 1 and Table 2). In cryopreservation of cell sheets, the basic composition is a freezing solution containing DMSO or EG [129,130,131,132,133,134,135,136,137,138,139]. Rewarming solutions containing sucrose are often used to thaw cryopreserved cell sheets. Oliva et al. examined vitrification and storage against oral mucosa epithelial cell sheets (CAOMECS) [129]. CAOMECS were cryopreserved by Vitrification procedures 1 and 2 in solutions as reported by Vitrification procedure 1 (Sheikhi et al.) [140], and Vitrification procedure 2 (Marco-Jimenez et al.) [141]. In contrast, Vitrification procedure 3 (Li et al.) [142] was cryopreserved CAOMECS in bulk. CAOMECS were prepared in transwells and placed in plastic containers. The plastic containers were placed in a liquid nitrogen freezer. The CAOMECS were preserved with the vitrification solution in the liquid nitrogen using Vitrification procedure 1 and 2. The cell sheets broke during the thawing process. In contrast, Vitrification procedure 3 was effective dry, in the absence of the vitrification solution, because the cell sheets were not damaged by the thawing process. Cryopreservation under liquid nitrogen using the vitrification solution were effective for the chondrocytes [132] and the myoblast sheets [133]. These cell sheets were placed in a bag held 1 cm above the surface of liquid nitrogen vapor (approximately −150 °C). The vitrified cell sheets can be stored for a long time and have potential for clinical application. Without vitrification or a controlled-rate freezer, MSCs [136] and the fibroblasts sheets [139] can maintain sheet morphology and function by slow freezing. Miyamoto et al. also succeeded in cryopreserving cell sheets such as primary rat hepatocytes, mouse embryonic fibroblasts (MEF), and mouse embryonic stem cells (ESCs) cultured on collagen vitrigel membranes [143]. These cells were used because cryopreservation of hepatocytes is difficult. Therefore, the recovery rate of adherent cells is significantly compromised by collection, freezing, thawing, and reattachment. Similar to the cellular- and tissue-based products described above, we used a cryopreservation solution containing 10% DMSO. The freezing procedure was performed in a controlled-rate freezer [108], and the storage temperature was maintained in liquid nitrogen. Compared with these cell sheets, the cryopreservation of collagen vitrigel adherent to MEF and mouse ESCs was more effective than that of primary rat hepatocytes.

## 6. Conclusions and Future Perspectives

Applications of various hydrogels will be considered in regenerative and cellular medicine [144,145]. Various cells have been encapsulated into hydrogels to construct desired tissues and organs. However, it is important to develop cryopreservation technology to utilize the created tissues and organs more effectively. 3D hydrogel cryopreservation, using alginic acid, synthetic polymers, and supramolecular gels, has been reported as effective [146]. Manferdini et al. focused on hydrogels for embedding the MSCs in vitro/ex vivo studies and reported a systematic review in an in vivo osteoarthritis (OA) model. [147]. The objective is cartilage regeneration in OA using hydrogels. In conclusion, the basic study results are positive, but cartilage strength and function need to be improved for clinical application. Next, we will discuss cell sheets from 3D hydrogel.

This review describes cell sheets, vitrified hydrogel membranes, and their cryopreservation applications in regenerative and cellular medicine. Synthetic polymers, thermoresponsive polymers, and ECMs such as collagen and gelatin, have been used to fabricate cell sheets. Numerous regenerative medical products for single-cell transplantation have been cryopreserved using DMSO. Similarly, cryopreservation of cell sheets is expected to further promote the medical industry. When using cell-sheet products, the highest priority should be the therapeutic effect on patients. If there is no difference in quality between frozen and unfrozen cellular products, such as artificial cell sheets, frozen cellular products may be chosen. In fact, ice-free vitrification cryopreservation methods were effective for the epithelial constructs (EpiDerm) from MatTek (www.mattek.com, accessed on 6 December 2022) for the testing of new chemicals and drug screening [148]. The safety and efficacy of cellular- and tissue-based products, as well as the manufacturing time, process, and cost, must be considered and judged as a comprehensive medical system. Because various cellular and tissue-based products (living and non-living cell products) have different uses, selecting the appropriate manufacturing and storage processes for cellular- and tissue-based products is essential, considering the adequacy of the facility’s equipment and medical system.

## Figures and Tables

**Table 1:** Preparation of cell sheets.

Cells	Origin	Species	Sheet Preparation	In Vivo Test	Year of Publication	Reference
Epithelial cells	Oral mucosa	Rabbit	Oral mucosal epithelium cells were cultured with MMC-treated NIH/3T3 feeder cells.	YES	2019	[129]
Epithelial cells	Foreskin keratinocytes	Human	Human epithelial cell sheets (ECSs) were cultured on plastic dishes.	YES	2021	[130]
Chondrocytes	Knee cartilage	Rabbit	Primary cultured cells derived from the knee cartilage were plated onto temperature-responsive culture dishes (UpCell^®^, CellSeed).		2020	[132]
Myoblast cells	Vastus medialis muscle	Human	Cell sheets consisting of myoblast cells were prepared using temperature-responsive culture dishes (UpCell^®^, CellSeed).	YES	2018	[133]
Mesenchymal stem cells (MSCs)	The bone marrow of femurs	Rat	Cell sheets consisting of mesenchymal stem cells were prepared using temperature-responsive culture dishes.	YES	2018	[136]
Fibroblasts	Tail skin	Mouse	Primary fibroblasts were inoculated on plastic dishes	YES	2022	[139]
Hepatocytes	Liver	Rat	Primary hepatocytes were inoculated onto collagen vitrigel membranes.		2009	[143]
Embryonic stem cells (ESCs)	Fertilized egg	Mouse	1st step: Primary mouse embryonic fibroblasts (MEF feeder cells) were inoculated onto UV-irradiated collagen vitrigels. 2nd step: A mouse ES cell culture was performed with mitomycin C-treated MEF feeder cell layers.		2009	[143]
Embryonic fibroblasts	Fetal tissues	Mouse	Primary mouse embryonic fibroblasts (MEF feeder cells) were inoculated onto collagen vitrigel membranes.		2009	[143]

**Table 2:** Freezing and thawing procedure of cell sheets.

Cells	Freezing Procedure	Thawing Procedure	Reference
Epithelial cells	**Vitrification of epithelial cell sheets** Vitrification Procedures (1, and 2) in solutions Vitrification Procedures (3) in bulk The plastic containers were placed in the liquid nitrogen freezer	**Stepwise** The cell sheets were thawed in four steps using the solutions (culture media, culture media supplemented with 0.2, and 0.1 M sucrose) at 37 °C.	[129]
Epithelial cells	**Programmed freezing** Epithelial cells were cryopreserved for storage in KGM and 10 μM Y27632 at 4 °C. Or cryochamber (Planer KRYO 10 Series III Freezer, UK) was run by the pre-set cooling program.	**Rapid thawing** Thawing was carried out rapidly by holding in air for 1 min to boil off any liquid nitrogen and swirling in a water bath at 40 °C.	[130]
Chondrocytes	**Vitrification of the chondrocyte sheets** The chondrocyte sheets in the circulating vitrification bag were held 1 cm above the surface of liquid nitrogen vapor (approximately −150 °C).	**Rapid thawing** The circulating vitrification bag was placed on a heating plate at 45 °C. The gel was placed on top of the cell sheet to thaw it rapidly.	[132]
Myoblast cells	**Vitrification of the myoblast sheets** The myoblast sheets were detached from the dish at 22 °C. The cell sheets were placed in thin polyethylene films and held 1 cm above the surface of liquid nitrogen vapor.	**Rapid thawing** Thin polyethylene films was placed on a heating plate at 37 °C. The gel was placed on top of the cell sheet to thaw it rapidly.	[133]
Mesenchymal stem cells (MSCs)	**Slow freezing** MSCs sheets were placed directly into a deep-freezer set at −80 °C.	**Rapid thawing** MSCs sheets were placed in a 37 °C water bath for rapid thawing until almost no ice was detectable.	[136]
Fibroblasts	**Stepwise** The fibroblasts sheets were placed into a 3D freezer (Koga Sangyo Co., Ltd.) at −35 °C for 20 min to freeze the cells and then transferred to a −80 °C deep freezer.	**Rapid thawing** The fibroblasts sheets were placed in a 37 °C water bath for rapid thawing until almost no ice was detectable.	[139]
Hepatocytes	**Programmed freezing** The hepatocytes sheets were placed in a controlled rate freezer (Kryo10, Planer) and frozen at a rate of 1 °C/min [108].	**Rapid thawing** To thaw the cells, 2 mL of culture medium warmed to 37 °C was added.	[143]
Embryonic stem cells (ESCs)	**Programmed freezing** The ESCs sheets were placed in a controlled rate freezer (Kryo10, Planer) and frozen at a rate of 1 °C/min [108].	**Rapid thawing** To thaw the cells, 2 mL of culture medium warmed to 37 °C was added.	[143]
Embryonic fibroblast	**Programmed freezing** The embryonic fibroblast sheets were placed in a controlled rate freezer (Kryo10, Planer) and frozen at a rate of 1 °C/min [108].	**Rapid thawing** To thaw the cells, 2 mL of culture medium warmed to 37 °C was added.	[143]

## Data Availability

Not applicable.
